# Longer First Introns Are a General Property of Eukaryotic Gene Structure

**DOI:** 10.1371/journal.pone.0003093

**Published:** 2008-08-29

**Authors:** Keith R. Bradnam, Ian Korf

**Affiliations:** Genome Center, University of California Davis, Davis, California, United States of America; University of Western Cape, South Africa

## Abstract

While many properties of eukaryotic gene structure are well characterized, differences in the form and function of introns that occur at different positions within a transcript are less well understood. In particular, the dynamics of intron length variation with respect to intron position has received relatively little attention. This study analyzes all available data on intron lengths in GenBank and finds a significant trend of increased length in first introns throughout a wide range of species. This trend was found to be even stronger when using high-confidence gene annotation data for three model organisms (*Arabidopsis thaliana*, *Caenorhabditis elegans*, and *Drosophila melanogaster*) which show that the first intron in the 5′ UTR is - on average - significantly longer than all downstream introns within a gene. A partial explanation for increased first intron length in *A. thaliana* is suggested by the increased frequency of certain motifs that are present in first introns. The phenomenon of longer first introns can potentially be used to improve gene prediction software and also to detect errors in existing gene annotations.

## Introduction

The discovery of RNA splicing in the late 1970s paved the way for our understanding of the exon-intron structure of eukaryotic genes [1]. Since then, data on intron and exon sequences have been steadily accumulating in databases such as GenBank [2]. While there have been several studies that have used data from multiple species to look at intron position [3]–[5], or intron length [6], [7], details concerning the length of introns at different positions within a gene are less well known. In particular, there is very little information concerning differences between introns that occur in coding sequences (CDSs) and introns that occur in the untranslated regions (UTRs) of a gene.

The few studies that have combined intron data from multiple species have found that the first intron is - on average - longer than later introns [8], [9], and that introns from the 5′ UTR region of a gene tend to be longer than introns from within the CDS or 3′ UTR [10], [11]. Other single-species studies have also confirmed the increased length of first introns in humans [12], [13], *Drosophila melanogaster* [14], mice [15], and *Arabidopsis thaliana* [16]. All of the aforementioned studies have been limited to studying low numbers of introns, or low numbers of species, or both. Hence the generality of increased first-intron length across all eukaryotes is still unclear.

One reason for distinguishing between introns that occur at different positions in a gene, is that introns from the 5′ proximal region of a gene (‘early’ introns) have been shown to have important functional properties, often relating to gene expression. The ability of an intron to enhance gene expression has been termed intron-mediated enhancement (IME) [17], though not all introns produce an IME effect and the sequence of any intron may be less important than its position [18]. Examples of early introns that have been shown to be essential for high expression, or required for normal expression, have been found in human [19], mice [20], *A. thaliana* [21], rice [22], [23] and *C. elegans* [24]. A sequence motif that might contribute to enhancing expression via IME has been predicted in *A. thaliana* and in rice [25].

There are other reasons to suggest that early introns might be functionally different from those that occur later on in the gene. First introns in humans (in comparison to later introns) contain fewer SNPs [26] and transcription factor binding sites [27], and have a reduced insertion frequency of SINE elements [28]. Comparisons between orthologous introns in different vertebrate species have also shown that the first intron (and the 5′ most region of the first intron in particular) tends to be the most conserved of all introns [15], [29]–[31]. Comparison of introns from different *Drosophila* species have also shown that first introns contain more conserved elements than later introns [14], [32]–[34].

In this study, we have looked at the issue of first intron length in several different ways. Firstly, and most importantly, we used information from GenBank to look at intron length data from a much larger number of species than has been previously studied. Secondly, by also studying high-quality gene annotations from three model organisms, it has been possible to systematically evaluate differences in intron length at different positions within a gene, ensuring that UTR introns are evaluated separately from CDS introns. By finding that a trend for increased length occurs in the vast majority of eukaryotic species, we establish that this is a general feature of eukaryotic gene structure. This phenomenon exhibits itself most strongly in the first intron of the 5′ UTR (though the first intron of the CDS also tends to be longer than downstream introns). Additionally, we find that in *A. thaliana*, this increased length can partly be accounted for by the presence of an IME motif that increases in frequency in early introns. Finally, we also show that because first introns tend to be longer, this information can be used to detect errors in existing gene annotations.

## Results

### Analysis of intron lengths using GenBank data

**Figure 1:**
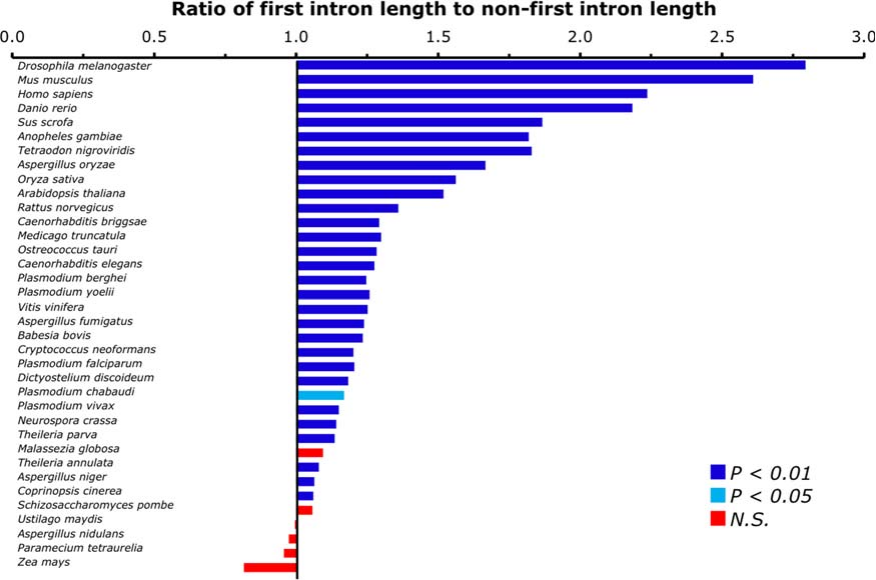
First introns are the longest introns in most species. Results shown for all species in GenBank release 164 which have at least 500 CDSs that specify multiple introns. Z-tests were used to determine significance and color denotes level of significance (see legend, N.S. = not significant).

Using data from GenBank, intron lengths were calculated for all available species with sufficient numbers of full-length CDSs (at least 500). For each species, the average length of all first introns was compared to the average length of all other introns (‘non-first’ introns). For 30 of 36 species with sufficient data, the average length of the first intron was seen to be significantly (Z test, *P*<0.05) longer than the average of the non-first introns (Figure 1). The most extreme case was observed in *D. melanogaster* where the mean length of the first intron is nearly three times as long as the mean length of later introns. Combined data from all species suggests that the first intron of the CDS tends to be ∼40% longer than later introns. Significantly longer first introns were found in species from diverse phylogenetic groups (including vertebrates, nematodes, insects, plants, and fungi), suggesting that this increased length is typical for all eukaryotic species.

**Figure 2:**
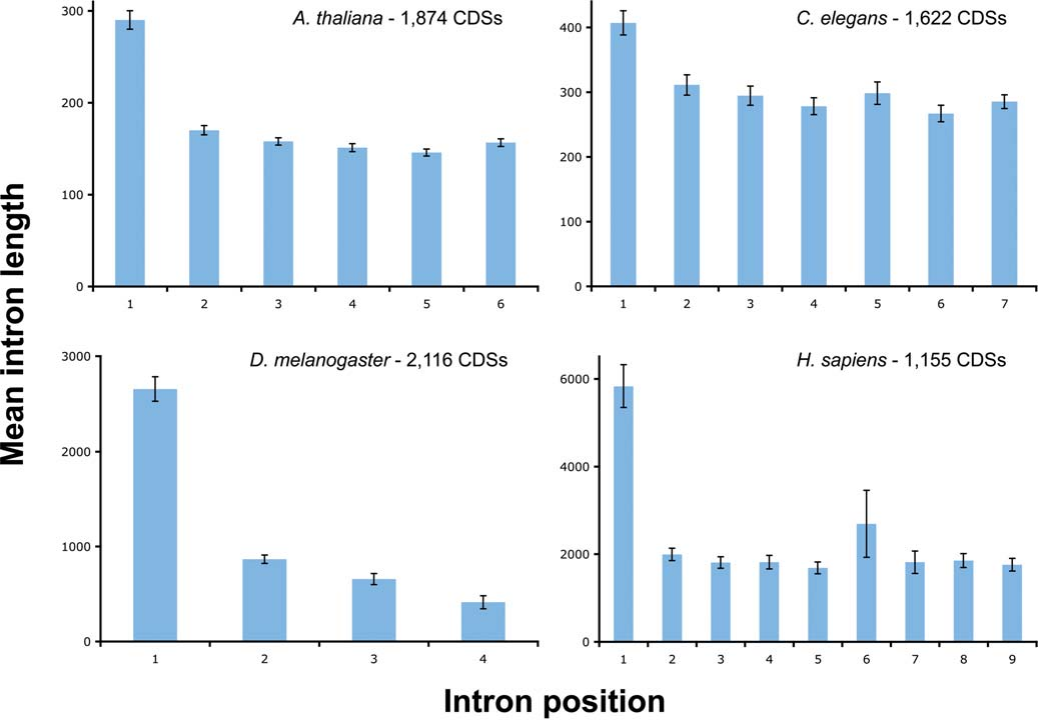
Intron size variation for selected species with different numbers of introns. Intron lengths are shown for species with CDSs that contain 4, 6, 7 or 9 introns (in *D. melanogaster, A. thaliana*, *C. elegans*, and *H. sapiens* respectively). Bars on graph show standard error of the mean. Numbers of CDSs used for each species are shown.

The trend for longer first introns was examined in more detail by partitioning the data for ‘non-first’ introns into separate classes, i.e. when 1st and 2nd intron lengths are compared in CDSs that have exactly the same number of introns. In total, 118 comparisons were made for species that have specific numbers of introns (excluding single intron CDSs). Of these, the majority (78%) showed a significant difference between the lengths of the 1st and 2nd intron (Z test: *P*<0.05, selected examples shown in Figure 2). In a small number of cases the 2nd intron was also significantly longer than introns that followed, though typically it was only the 1st intron that could be distinguished from other introns (data not shown). Significant differences between 1st and 2nd intron lengths occurred in CDSs that contained as few as 2 introns (e.g. mean lengths of 1st and 2nd introns in *Aspergillus niger*: 99 nt vs 91 nt, *P*<0.01), or as many as 20 introns (e.g *Oryza sativa*: 554 nt vs 344 nt, *P*<0.01).

### Analysis of introns using high-confidence gene annotations

**Figure 3:**
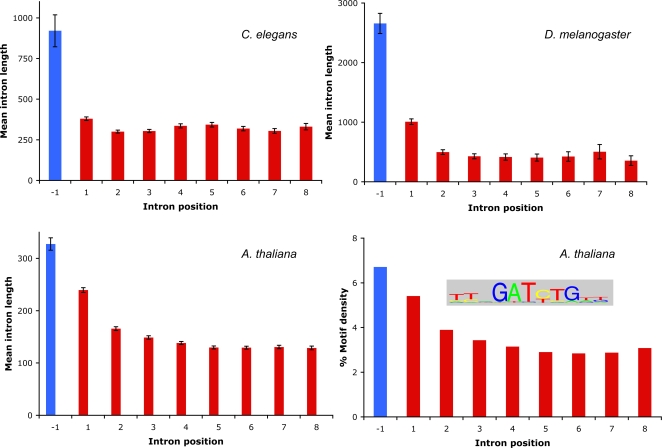
Intron length variation in three model organisms. Mean intron length is calculated for the first intron in the 5′ UTR (position −1, in blue) and for the first eight introns of the coding sequence (in red) for three named species. Error bars indicate standard error of the mean. Bottom right panel shows the occurrence of a potential IME motif (pictured) in *A. thaliana* introns. %Motif density is calculated by concatenating together all introns in each category, and then calculating what fraction of the total sequence is occupied by the motif.

A major limitation to using CDS data from GenBank is that this data excludes any introns that might be located in the 5′ UTR of the gene. Therefore, many ‘first’ introns in the GenBank data may actually be the second or third intron in the gene. Another problem is that GenBank contains redundancy; identical intron sequences may be present in multiple entries as alternative splice variants and/or as sequences from different strains of the same species. To counter these problems, we used intron data from sets of high-confidence gene annotations in three model organism species (taking just one isoform per locus). These annotations were all supported by cDNA evidence and revealed introns in the 5′ UTR of many coding transcripts (the proportion of genes with 5′ UTR introns was 23%, 15%, and 7% for *D. melanogaster*, *A. thaliana*, and *C. elegans* respectively). Analysis of these introns confirmed the prior observation of longer first introns and also revealed that while the first intron of the coding region remains significantly longer than downstream coding introns, it is the first intron of the 5′ UTR, if present, that is the longest intron within a transcript (Figure 3). Because most genes do not have 5′ UTR introns, we looked for differences between first introns of the CDS in genes that either possess or do not possess an upstream 5′ UTR intron. The first intron of the CDS was found to be longer in genes which do not have a prior intron in the UTR compared to those that do. For example, in *A. thaliana* the mean length of the first intron from all CDSs was 239 nt. In the 77% of CDSs without an upstream UTR intron the length increases slightly to 248 nt, and in the 23% of CDSs that do have a 5′ UTR intron the length falls to 183 nt.

An unusual feature of the *C. elegans* genome is that many genes (∼15%) are arranged in operons [35]. Therefore, the first intron of a downstream gene in an operon, will not be the first intron in the transcript. To investigate introns in operons, we divided 1st introns into those that are from the first gene in an operon, those that are from downstream genes in operons, and those that are from genes not in operons. The mean lengths of these three groups of 1st introns are 230 nt, 318 nt, and 458 nt respectively. This shows that introns within operons tend to be shorter than the average *C. elegans* intron (344 nt), with the very first intron in the operon transcript being notably shorter than the average.

### Early introns in *Arabidopsis* contain a higher frequency of IME motifs

Using motif prediction software, a motif has been discovered in *A. thaliana* introns that is postulated to be involved in the process of intron mediated enhancement (IME) of gene expression [25]. Because this motif preferentially occurs in early introns, the increased lengths of first introns might simply be due to an enrichment of this motif. This hypothesis has some support in *A. thaliana*; the aforementioned IME motif accounts for a greater proportion of early introns than later introns (Figure 3). We found that this motif more than doubles in frequency in the first intron of 5′ UTRs when compared to downstream introns. Furthermore, it was present (in at least one copy) in 74% of first introns from the 5′ UTR compared to only 27% of the fifth introns from the CDS. However, this motif does not account for all of the additional length of first introns; removing the motif from the first introns of 5′ UTRs reduces their average length from 328 nt to 306 nt, which is still substantially longer than downstream introns.

### Using intron length information to detect errors in gene annotation

**Figure 4:**
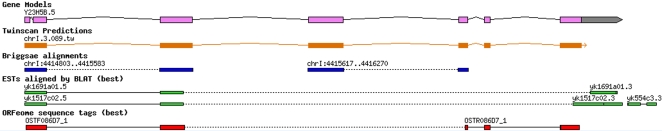
Incorrect *C. elegans* gene annotation determined by inspection of intron lengths. This gene prediction contained an incorrect in-frame intron sequence in the first exon. Transcript evidence, homology evidence from *C. briggsae*, and an alternative gene prediction (Twinscan) suggested that the first intron is an annotation error. Image taken from Genome Browser display of WormBase release WS180 (http://ws180.wormbase.org).

We investigated whether knowledge that introns at different positions tend to be different lengths, could be useful in finding mistakes in publicly available gene annotations. Potential candidate annotation errors were identified in *C. elegans* by looking for genes with a shorter-than-average first intron followed by a much longer-than-average second intron. Twenty of these examples were inspected and four of them (20%) were revealed to be annotation errors. In some cases the problem with the annotation was obvious as there was clear supporting evidence that the first intron should be deleted from the annotation (Figure 4). These curation errors were reported to the WormBase database and annotations have subsequently been corrected.

## Discussion

### Intron data reveal a major pattern to eukaryotic gene structure

While there are many individual examples of genes with short first introns, the dominant trend appears to be that, on average, it is the first intron that is the longest intron in a gene. Utilizing data from GenBank, we show that the first intron is the longest intron for the majority (32 of 36) of species (Figures 1 and 2). While there are caveats in relying on GenBank data - it contains many annotations deriving from *in silico* predictions that have little or no supporting experimental evidence - it has been shown that the lengths of exons and introns from several whole genome annotations are in good agreement with lengths derived from EST-confirmed genes [7]. We therefore believe that for species with sufficient data, GenBank is a useful and reliable source of intron lengths. Of the six species which did not show significantly longer first introns, only four showed the opposite trend of having shorter first introns. Two of these four species still had a majority of genes with first introns which are longer than the second intron (the lack of significance arises from pooling all non-first introns together). One of the remaining two species is *Paramecium tetraurelia*. This species is unusual in only possessing very short introns that have little variation in length [36]. The average length of first and non-first introns are very similar in this species (25.3 nt vs 26.4 nt). The remaining species (*Zea mays*) was the only one that was found to have notably shorter first introns. This is likely to be caused by a small sample size (N = 645 CDSs) which also features several genes which are overrepresented due to being sequenced multiple times from different strains (one gene alone represents over 10% of the total dataset).

Any limitations in the GenBank data can be overcome by focusing on high-quality annotations in which only one isoform is represented from each locus, and in which introns from 5′ UTRs can be clearly distinguished from introns in the CDS. Such data (Figure 3) confirms the increased length of the first CDS intron but also points to an even more pronounced trend for the longest introns occurring in the 5′ UTR. While the first intron tends to be longest regardless of whether it is in the 5′ UTR or in the CDS, there is still a distinction in that the first introns of the 5′ UTR are much longer than the first introns of genes that do not possess 5′ UTR introns. Given that 5′ UTR introns are relatively rare it will be of interest to see whether the subset of genes that do possess them have any functional characteristics in common.

One notable exception to the pattern of increased first intron length was found in the subset of *C. elegans* genes that occur in operons. First introns in operons are surprisingly shorter than expected and the shortest introns are those that occur first in the operon transcript. It is unclear why introns in operons differ in this way, and this suggests that the processes that contribute to increasing first intron length might act differently in operons (if at all). Operons are known to differ in other ways, containing genes that are known to be biased towards certain essential functions such as transcription and translation [37]. Overall, the results of this study supports and extends the previous studies of intron length that have also found evidence of longer first introns [8]–[16], and apart from the exception of *C. elegans* operons, we believe that this work underlines the generality of this phenomenon across phylogenetically diverse species.

### Uses of intron length information

Genome annotation requires sophisticated computer tools to integrate various strands of experimental and computational evidence. Computational gene-finders integrate many known properties of real genes (coding bias, splice site consensus sequences etc.), and can include models to reflect different classes of intron length (e.g. [38]). However, we are not aware of any programs that weight gene predictions according to the relative intron lengths at different positions in the gene. The preliminary results for *C. elegans* suggest that this is a justifiable method for finding errors in annotations (Figure 4). As it is known that long introns can be problematic for gene finding programs [39], it may prove useful for gene finding algorithms to make allowances for the increased length of first introns.

### Why are first introns longer?

A simple hypothesis to explain the increased length of first introns is that these introns contain functional elements that are present in addition to what might be regarded as ‘normal’ intron structure. There is evidence that this might be the case for why the first introns of mouse and rat are longer [15]. The authors of that study postulate that an increase in the presence of functional elements in first introns may be involved in controlling gene expression. A potential IME motif that might enhance gene expression has been predicted from an analysis of early intron sequences in *A. thaliana* and rice [25]. Longer introns have also previously been observed to be more common in highly expressed genes in these two species [40], [41], though the authors also report higher expression being associated with shorter introns in other species (see also [42]). This potential IME motif has also been detected in the early introns of grape and poplar but not in *C. elegans* or *D. melanogaster* (unpublished data), suggesting that it might be a motif common to plants. In our analysis, we find that while this 11 nt motif is present at a background level in all *A. thaliana* introns, it occurs far more frequently in early introns.

Given the numerous reports that have shown that first introns tend to be under more selective constraint (see Introduction), it seems likely that there could be many different sequence elements present in these early introns. If such functional elements acted as binding sites for proteins (or RNAs), then it may be important to have adequate spacing between elements to allow multiple proteins to bind without causing interference. This raises the possibility that increased intron length may also be partly due to accumulation of additional ‘filler’ DNA. Identifying the range and extent of sequence elements in first introns may not be easy. Some elements may only be conserved in specific taxonomic groups (as may be the case with the potential IME motif in plants), and some elements may serve functions unrelated to gene expression. The first intron of the *D. melanogaster Adh* gene contains a hairpin structure that appears important for normal splicing activity of that gene [43]. Distinguishing between elements that may have different functions, and fully delimiting the extent of motifs that might be responsible for increased first intron length will require more experimental work. Systematic deletion studies of first introns and generation of synthetic first introns will hopefully illuminate our understanding of why first introns are longer.

## Materials and Methods

### GenBank data

A perl script was written to extract data from release 164 of the GenBank database [2]. Only sequences with a molecule type of DNA from the INV, MAM, PLN, PRI, ROD, VRT, and HTG divisions and sequences in the Whole Genome Shotgun (WGS) directory were utilized. Intron lengths were inferred from the coordinates of coding sequence (CDS) features which specify the locations of protein-coding regions within a gene. Partial CDSs (where the translation start and/or stop sites are not known) were excluded as were CDS features that spanned multiple GenBank entries. Any CDSs which contained introns of less than 20 nucleotides in length were also ignored. This filtering produced a set of nearly two million CDSs from almost 3,000 species.

For each species, the mean length of introns at different positions within the CDS was calculated in two different ways. First, CDSs were separated into different classes - based on how many introns they contained - and mean intron length was calculated at each intron position within each class (with a requirement of at least 100 introns in each position). Secondly, all introns were classified as either ‘first intron’ or ‘non-first intron’. Additional filtering restricted this data to species with at least 500 CDSs in GenBank, and excluded any single-intron CDSs. Mean intron lengths were compared by use of a Z-test.

### High-confidence genome annotation data

Details of the genome annotations of *A. thaliana*, *C. elegans*, and *D. melanogaster* were extracted from their respective model organism databases TAIR, WormBase and FlyBase [44]–[46]. Downloaded GFF and DNA files from these databases were parsed using perl scripts to to obtain length and sequence information from introns located at all positions within a transcript. Differences between the content of the three databases meant that each species was treated differently in order to only extract intron details from genes that were best supported by transcript data (cDNAs, ESTs). For genes with multiple transcript isoforms, only the first named or numbered transcript was retained.

### *Arabidopsis thaliana*

- Used TAIR release 7
- Only retained transcripts with annotated 5′ and 3′ UTRs
- Removed genes that are 95% identical to others over 400 nt
- Removed genes with introns <60 bp or >1000 bp, or CDS lengths <50 amino acids
- Final data set: 13,163 transcripts containing 59,260 introns

### *Drosophila melanogaster*

- Used FlyBase release r5.7
- Only retained transcripts that had a FlyBase evidence score of at least 8
- Final data set: 7,486 transcripts containing 19,772 introns

### *Caenorhabditis elegans*

- Used WormBase release WS190
- Only kept transcripts deriving from genes with a WormBase ‘Confirmed’ designation
- Final data set: 6,232 transcripts containing 30,916 introns

Average intron lengths were calculated for each intron position, pooling data from transcripts with different numbers of introns. I.e. the 2nd intron of a gene with just two introns was included with the 2nd intron of a gene with ten introns. A small proportion of 5′ UTRs contained two or more introns, but only the first 5′ UTR intron was considered in this analysis.

### Motif analysis

We compared the sequences of *A. thaliana* introns in the high-confidence data set to a previously discovered *A. thaliana* sequence motif believed to be involved in IME [25]. A perl script was written to calculate the amount of motif in all intron sequences at each intron position. This was done by using a sliding-window across every position in each intron (window size = 11 nt, step size = 1 nt). For each observed nucleotide in the sliding-window, we divided the corresponding frequency of that nucleotide in the motif by the background frequency of the same nucleotide in all intron sequences. The logarithms of these scores were calculated and summed across all positions in the window (producing a log-odds score). Positive log-odds scores denote that the window of intron sequence resembles the motif, and we chose a log-odds threshold of 3 for the purpose of counting motifs. Because multiple occurrences of this motif can overlap (the motif tends to start and end with a TT dinucleotide), we preferred a measure of ‘motif density’ rather than ‘motif count’. For each intron position, motif density was calculated as the total number of bases that were occupied by the motif divided by the total length of all introns at that position.
